# Characterization of the promoter region of the murine *Catsper2* gene

**DOI:** 10.1002/2211-5463.13518

**Published:** 2022-11-17

**Authors:** Andrea del Pilar Contreras‐Marciales, Sergio Federico López‐Guzmán, María Luisa Benítez‐Hess, Norma Oviedo, Javier Hernández‐Sánchez

**Affiliations:** ^1^ Departamento de Genética y Biología Molecular Centro de Investigación y Estudios Avanzados del Instituto Politécnico Nacional (CINVESTAV) Ciudad de México Mexico; ^2^ Unidad de Investigación Médica en Inmunología e Infectología, Centro Médico Nacional, La Raza Instituto Mexicano del Seguro Social Ciudad de México Mexico

**Keywords:** *Catsper2*, CREM, CTCF, spermatogenesis, transcriptional regulation

## Abstract

CATSPER2 (Cation channel sperm‐associated protein 2) protein, which is part of the calcium CATSPER channel located in the membrane of the flagellar principal piece of the sperm cell, is only expressed in the testis during spermatogenesis. Deletions or mutations in the *Catsper2* gene are associated with the deafness‐infertility syndrome (DIS) and non‐syndromic male infertility. However, the mechanisms by which *Catsper2* is regulated are unknown. Here, we report the characterization of the promoter region of murine *Catsper2* and the role of CTCF and CREMτ in its transcription. We report that the promoter region has transcriptional activity in both directions, as determined by observing luciferase activity in mouse Sertoli and GC‐1 spg transfected cells. WGBS data analysis indicated that a CpG island identified *in silico* is non‐methylated; Chromatin immunoprecipitation (ChIP)‐seq data analysis revealed that histone marks H3K4me3 and H3K36me3 are present in the promoter and body of the *Catsper2* gene respectively, indicating that *Catsper2* is subject to epigenetic regulation. In addition, the murine *Catsper2* core promoter was delimited to a region between −54/+189 relative to the transcription start site (TSS), where three CTCF and one CRE binding site were predicted. The functionality of these sites was determined by mutation of the CTCF sites and deletion of the CRE site. Finally, ChIP assays confirmed that CREMτ and CTCF bind to the *Catsper2* minimal promoter region. This study represents the first functional analysis of the murine *Catsper2* promoter region and the mechanisms that regulate its expression.

## Graphical abstract

CATSPER2 is necessary for sperm hyperactivation and male fertility. Here, we report the characterization of *Catsper2* promoter region and identify a CpG island *in silico*. ChIP‐seq data analysis revealed that histone marks H3K4me3 and H3K36me3 are present in the promoter and body of the *Catsper2* gene. Finally, CTCF and CREMτ bind to the *Catsper2* core promoter and regulate its expression.

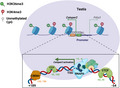

**Abbreviations**
CATSPER: cation channel of sperm
CATSPER2: cation channel sperm‐associated protein 2
CRE: cAMP response element
CREM: cAMP responsive element modulator
CREMτ: cAMP responsive element modulator isoform τ
CTCF: CCCTC‐binding factor
EPD: eukaryotic promoter database
WGBS: whole genome bisulfite sequencing

CATSPER2 (Cation channel sperm‐associated protein 2), which has a sperm‐specific expression, is a mouse (*Mus musculus*) 588 a.a. protein that shares 67% identity with human CATSPER2. Along with other three trans‐membrane subunits (CATSPER1,3 and 4), five auxiliary subunits (CATSPER β, γ, δ, ε, ζ) and an EF‐hand calcium‐binding domain‐containing protein 9 (EFCAB9) compose the calcium channel located in the membrane of the flagellar principal piece of spermatozoid. Disruption of any CATSPER subunit produces male infertility by disturbing calcium concentration and preventing sperm hyperactivation [1, 2, 3, 4, 5, 6, 7]. *Catsper* gene expression varies through spermatogenesis; while *Catsper1*,*3* and *4* are expressed in spermatids at late stages, *Catsper2* is found in primary spermatocytes at earlier stages, suggesting an essential role for *Catsper2* in the regulation of the other *Catsper* members [1, 2, 4, 5, 6, 8, 9, 10]. Interestingly, deletions or mutations in *Catsper2* gene have been associated with patients with the deafness‐infertility syndrome (DIS) or non‐syndromic male infertility, highlighting the importance of *Catsper2* gene in human male fertility. [11, 12, 13, 14, 15]. Even though the relevance of *Catsper2* in male fertility is well established, the mechanism by which this gene is transcriptionally regulated is unknown. We previously reported that the cAMP Responsive Element Modulator τ isoform (CREMτ) regulates the *CATSPER1* promoter activity *in vitro* [16]. CREMτ is a testis‐specific isoform of the *Crem* gene that contains a basic region and leucine zipper domains for CRE site recognition and DNA binding, in addition to 3 activation domains (Q1, Q2 and KID) for transcriptional activation [17, 18, 19]. *Cremτ* knockout mice exhibit male infertility due to post‐meiotic cell arrest and increased apoptotic germ cells [20]. Also, up to 101 genes involved in spermatogenesis were downregulated in *Cremτ* knockout mice [21]. On the other hand, CCCTC‐Binding Factor (CTCF) is a ubiquitous transcription factor relevant to male fertility that comprises 11 Zinc fingers for DNA‐binding [22]. *Ctcf* conditional knockout male mice are unable to procreate offspring, have smaller testes than wild‐type mice, and lots of genes, including *Catsper2* were downregulated in these mice as shown by microarray assay [23]. Altogether, these data suggest that CREMτ and CTCF play a significant role in the transcriptional regulation of *Catsper2*. In this study, we report the functional analysis of the murine *Catsper2* promoter region. The results indicate that CREMτ and CTCF transcription factors transactivate *Catsper2* promoter through the binding to specific sites of the core promoter. Regulation of *Catsper2* promoter by these factors may be crucial in mediating its specific expression in male germ cells.

## Materials and methods

### Animals

All the animals used in this work were bred according to the animal protocol 0113‐14 approved by the Internal Committee for the Care and Use of Laboratory Animals (CICUAL). CD1 mice were kept in polysulfonate cages with male mice, maintained in a 12 h light/dark cycle and provided with food and water *ad libitum*. CD1 mice were provided by the Unit for Experimentation and Production of Laboratory Animals (UPEAL) of the Center for Research and Advanced Studies (CINVESTAV), Mexico City, Mexico. Surgery was performed in accordance with the Ethical Guidelines and Procedures from the CICUAL. First, mice were euthanized by cervical dislocation, lower abdomen was then opened with surgical scissors and testes were exposed by pulling the epididymal fat. Later, testes were extracted, and tunica albuginea was removed with bistoury and finally washed with cold 1× PBS.

### RNA extraction and reverse transcription polymerase chain reaction (RT‐PCR)

For cloning *CREMτ* transcription factor, RT‐PCR was performed from mouse testis; total RNA was extracted using the TRIzol® reagent (Invitrogen, Waltham, MA, USA), according to the manufacturer's instructions. The cDNA was prepared from total RNA using a High‐Capacity cDNA Reverse Transcription Kit (Applied Biosystems, Waltham, MA, USA). PCR reactions were conducted with 1 U Phusion DNA Polymerase (New England Biolabs, Ipswich, MA, USA), 1× buffer HF, 200 mm dNTPs, 0.2 μm primers (Table 1), and 1 μL cDNA. The 1134 bp fragment of the *CREMτ* open reading frame (ORF) was cloned into pjet 2.1 blunt vector and then subcloned into pcDNA3 expression vector (pcDNA3‐CREMτ).

**Table 1:** Primers used in this work.

Reaction	Name	Sequence (5′–3′)	Product length (bp)	Templates
Transcription Factor cloning	CREMτ Fwd	ggatccaataatgagcaaatgtggcagg	1134	Testis total RNA
	CREMτ Rev	ctcgaggcaactgtacatgctgtaatcag		
Promoter cloning	Cats2F/−1309	tgaagcttgtttgaattaagggtcattgtg	1598	Genomic DNA
	Cats2R/+289	tgaagcttctgacatttggaaaacgtgtc		
	Cats2F/−509	tgaagcttggcctgctagatagtggag	798, 698	
	Cats2F/−215	tgctcgaggagaaatgagatgggagag	504	
	Cats2F/−54	tgctcgagaacagctggtaactgcc	343, 243	
	Cats2F/+3	tgctcgaggcgaacgcccg	287	
	Cats2R/+189	tgaagctttcgcgggcctcacttcc		
Sequencing constructions	RVPrimer3	ctagcaaaataggctgtccccag		
	Luc2Rev	gtcccgtcttcgagtgggtag		
	CMV Fwd	cgcaaatgggcggtaggcgtgt		
	BGH Rev	cctcgactgtgccttcta		
Mutagenesis	mCTCF1 Fwd	gcggaacagctggtattcttattgtggcgggcgcgcggg		pCat2‐243 plasmid
	mCTCF1 Rev	cccgcgcgcccgccacaataagaataccagctgttccgc		
	mCTCF2 Fwd	gggcgcgcggggcgattcttattgttcgcttaggcgaacgc		
	mCTCF2 Rev	gcgttcgcctaagcgaacaataagaatcgccccgcgcgccc		
	mCTCF3 Fwd	agccggagcggcccttattcttgcccgctggggaggag		
	mCTCF3 Rev	ctcctccccagcgggcaagaataagggccgctccggct		
	ΔCREMτ Fwd	gccggcgcggaaggcggaggtgagtgggcggaagtgaggc	229	
	ΔCREMτ Rev	gcctcacttccgcccactcacctccgccttccgcgccggc		

### Molecular cloning and sequence analysis

The Eukaryotic Promoter Database (EPD, https://epd.epfl.ch//index.php) was used to predict the putative promoter region of the murine *Catsper2* gene. The gene‐specific primers (Cats2F/−1309 and Cats2R/+289, Table 1) were designed to amplify a putative 1598‐bp promoter region of the murine *Catsper2* gene (NCBI accession NC_000068.8 from 121245582 to 121243985) including the transcriptional start site (+1). PCR amplification was performed using genomic DNA from CD1 mouse testis as a template and Phusion DNA Polymerase (New England Biolabs, Ipswich, MA, USA). The 1598‐bp fragment of the putative *Catsper2* promoter was cloned into pjet 1.2 blunt vector. This region was used as a template for amplifying a 798‐bp fragment containing a shorter promoter region (primers Cats2F/−509 and Cats2R/+289, Table 1) which was also cloned into pjet 1.2 blunt vector. Both fragments (1598‐ and 798‐bp) were subcloned in sense (pCat2‐1598 and pCat2‐798) and antisense (pCat2‐1598AS and pCat2‐798AS) directions using two flanking HindIII sites into the *Photinus* luciferase reporter vector pGL4.10. The potential TF‐binding sites were identified using promo (http://alggen.lsi.upc.es/cgi‐bin/promo_v3/promo/promoinit.cgi?dirDB=TF_8.3) and tf‐bind (https://tfbind.hgc.jp) and CpG islands were predicted with methprimer (http://www.urogene.org/methprimer/). Sequence alignment was performed using clustalx v2.1 (http://www.clustal.org/clustal2/).

### ChIP‐seq data analysis

ChIP‐seq data collected from the NCBI GEO and the sequence read archive (SRA) databases (Table 2) were analyzed as previously described [24] with minor modifications. Short read sequences were extracted in fastq format from the downloaded data in SRA format using sratoolkit (version 2.10.8). The extracted sequences were mapped to the mouse reference genome (mm10) with bowtie2 (version 2.3.5.1) [25]. The alignment parameter was set to default. ChIP‐seq peaks were identified using macs2 [26] with – nomodel parameter. Then, the ChIP‐seq enrichment calculations were performed with bamCompare from the deeptools suite (version 3.3.2) [27]; the normalization method was Bins Per Million mapped reads (BPM) with a bin size of 20 (‐‐extendReads 150). The resulting comparison values for *Catsper2* gene and promoter region were plotted. Only the run with a higher number of spots for the samples with duplicate experiments was analyzed.

**Table 2:** ChIP‐seq data.

Histone mark	Cells/tissue	Access number	Database	Ref.
H3K4me3	Spermatocyte pachytene	SRX336649 (A) SRX336651 (I)	SRA	[28]
	Round spermatids	SRX336652 (A) SRX336654 (I)	SRA	[28]
	Testis 8 weeks	GSM1000079 (SRR566800) (A) GSM1000203 (SRR566962) (I)	GEO	[29]
	Liver 8 weeks	GSM769014 (SRR317233) (A) GSM769034 (SRR317274) (I)	GEO	[29]
H3K36me3	Testis 8 weeks	GSM1000067 (SRR566775) (A) GSM1000203 (SRR566962) (I)	GEO	[29]
	Liver 8 weeks	GSM1000151 (SRR566943) (A) GSM769034 (SRR317274) (I)		

(A), Antibody sample; (I), Input sample.

### Whole genome bisulfite sequencing data analysis

To analyze and compare the methylation status of the CpG island found *in silico*, whole‐genome bisulfite sequencing (WGBS) data were collected from ENCODE project in BigWig format [30, 31]. The methylation status for *Catsper2* promoter region in spermatogenic cells and control tissues were plotted.

### Construction of *Catsper2* promoter deletions

Deletions of the 5′ and 3′ ends of the mouse *Catsper2* promoter were constructed using primers: Cats2F/−509_Cats2R/+189, Cats2F/−215_Cats2/+289, Cats2F/−54_Cats2R/+289, Cats2 P4/Cats2 P‐Rev2 (−54/+189) and Cats2 P5/Cats2 P‐Rev (+3/+289). PCR fragments were generated using pCat2‐798 as a template, cloned into pjet 1.2 blunt vector, and subcloned in sense containing XhoI and HindIII sites into pGL4.10 vector. These plasmids were named pCat2‐698, pCat2‐504, pCat2‐343, pCat2‐243 and pCat2–287, respectively. The integrity and orientation of all constructions were confirmed by automatic DNA sequencing with RVPrimer3 and Luc2Rev primers (Table 1).

### Site‐directed mutagenesis

Predicted CREMτ and CTCF binding sites from the pCat2‐243 construct were mutated using the Quick‐Change Site‐Directed Mutagenesis Kit (Stratagene, La Jolla, CA, USA) with the appropriate primers (Table 1). PCR was performed under the following conditions: 94 °C for 5 min; 40 cycles of 94 °C for 30 s, 68 °C for 6 min; and finally, 68 °C for 10 min. The reaction product was treated with DpnI and transformed into *Escherichia coli* DH5α competent cells and analyzed by sequencing.

### Cell culture and transfection

Type B spermatogonia GC‐1 spg and mouse Sertoli MSC‐1 cell lines were cultured in DMEM (Sigma, St. Louis, MO, USA) supplemented with FBS 10%, Penicillin (5 U·mL^−1^) and Streptomycin (5 mg·mL^−1^; 1%) and incubated at 37 °C in a humidified atmosphere with 5% CO_2_. Cells grown to 80% confluency were seeded in 24 well plate (1 × 10^5^ cells per well) 24 h before transfection. Cell transfections were performed with the K2® Transfection System (Biontex, München, Germany) according to the manufacturer's protocol with 500 ng of *Catsper2* reporter plasmids. Co‐transfections were made using 250 ng of *Catsper2* promoter plasmids and 250 ng of CREMτ‐expressing (pcDNA3‐CREMτ) or CTCF‐expressing vector (pcMV6, OriGene, Rockville, MD, USA) and 0.4 ng of pRL‐CMV normalization vector. The pGL4.10‐CMV and empty pGL4.10 vectors were used as positive and negative controls, respectively.

### Luciferase assay

Two days (48 h) after transfections, cells were washed with sterile 1× PBS and lysed, and the transcriptional activity was quantified using the Dual‐GLO Luciferase Assay System (PROMEGA, Madison, WI, USA) as described in the manufacturer's protocol. The relative luciferase activities were measured with a Fluoroskan Ascent reader (Thermo Fisher Scientific, Waltham, MA, USA). Luciferase activity was normalized to pRL–CMV activity and expressed as fold change relative to the activity of empty vector pGL4.10 (defined as 1). Data are presented as mean ± SEM of three independent experiments in triplicate.

### Immunodetection of CREMτ and CTCF

Total protein was extracted from GC‐1 spg cells 48 h after transfection with RIPA buffer containing 1 mm PMSF and Complete 1× (Roche, Basel, Switzerland), and protein concentration were determined by Bradford method (BioRad, Hercules, CA, USA). Protein samples were separated by 10% SDS/PAGE and subsequently transferred to a Polyvinylidene difluoride membrane (PVDF; BioRad). Membranes were blocked with 5% ProPure milk (Amresco, Solon, OH, USA) in TBS‐T buffer (Tris‐buffered saline, 0.1% Tween 20) 1 h and then incubated overnight with antibody diluted in blocking buffer including Santa Cruz (Dallas, TX, USA) antibodies anti‐CTCF (sc‐271474) anti‐CREM (sc‐39426), anti‐MYC (sc‐40), 1 : 500 dilution and anti‐GAPDH (sc‐365062), 1 : 1000 dilution. Finally, for CREMτ assay, membranes were incubated with a rabbit Anti‐Mouse IgG Polyclonal Antibody, Horseradish Peroxidase (HRP) conjugated (Cat. No. 61‐6520; Invitrogen, Waltham, MA, USA) 1 : 3000 dilution in blocking buffer for 1 h. The peroxidase was developed using Immbilon Forte Western HRP substrate (WBLUF0100; MilliporeSigma, St, Louis, MO, USA). For CTCF assay, membranes were incubated with AP‐Goat anti‐Mouse IgG (H + L; Cat. No. 62‐6522; Invitrogen) 1 : 1000 dilution in blocking buffer for 1 h and the bands were developed with BCIP^®^/NBT Liquid Substrate System (Cat. No. B1911; Sigma).

### Chromatin immunoprecipitation (ChIP) assay

Protein‐DNA complexes were prepared from the testis or liver of adult mice (*n* = 3). The ChIP assay was performed according to the X‐ChIP protocol from the Abcam protocols book (https://docs.abcam.com/pdf/misc/abcam‐protocols‐book.pdf) with minor modifications. Briefly, the complexes were preserved by fixing the cells in 1% formaldehyde for 30 min at room temperature and neutralization with glycine. The cells were then washed, lysed, and sonicated to shear DNA into fragments of 200–1000 bp; 10% was saved as input. DNA‐protein complexes were immunoprecipitated with 2 μg CTCF (sc‐271514X; Santa Cruz, Dallas, TX, USA), CREM (sc‐39426; Santa Cruz), or irrelevant IgG MHC Class II (14‐5321‐81; eBioscience, Waltham, MA, USA) antibody overnight at 4 °C. The immune complexes were isolated with protein G agarose beads (sc‐2002; Santa Cruz), eluted with salts, and finally, the crosslinking was reversed and digested with proteinase K. The DNA was then purified with the phenol‐chloroform method. The binding of transcription factors to the *Catsper2* promoter was assessed by standard PCR using the primers listed in Table 1. The IgG sample and liver tissue were used as negative controls. Densitometric analysis of CTCF and CREM ChIP–PCR signals was performed using imagej software (https://imagej.nih.gov/ij/index.html), quantified as electronic density (pixels) and plotted as IgG relative expression. Data are presented as mean ± SEM of three independent experiments.

### Statistical analysis

Statistical analysis was performed using graphpad prism 6 (GraphPad, San Diego, CA, USA). One‐way ANOVA and paired Student's *t*‐test were used to determine significant differences. Data show mean ± SEM (*n* = 9) from three independent experiments performed in triplicate; *P* < 0.05 was considered statistically significant.

## Results

### Identification of the murine *Catsper2* promoter and its regulatory elements

**Fig. 1:**
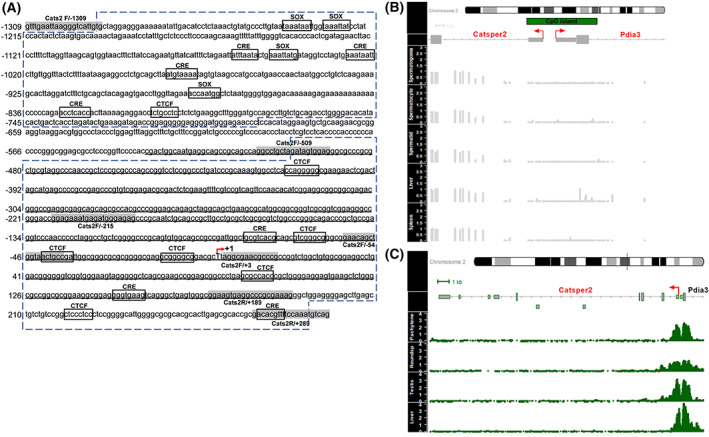
Regulatory elements of the mouse *Catsper2* promoter. (A) Red arrow marks the transcription start site designated as +1. Putative promoters predicted with eukaryotic promoter database are enclosed with blue dashed lines and primer alignment sequences for plasmid constructs are highlighted in gray. A predicted CpG island is underlined in black, the putative transcription factor sites are boxed, and their corresponding names are shown above. (B) Methylation status of the CpG island found at the mouse *Catsper2* promoter based of WGBS data analysis. The WGBS data for spermatogonias, spermatocytes, spermatids, liver, and spleen were obtained from ENCODE and plotted in the promoter region of murine *Catsper2* gen; scale denotes unmethylated CpG sites as 0 and fully methylated sites as 1, represented in gray color. (C) Histone H3K4 trimethylation pattern at the mouse *Catsper2* locus based on ChIP‐seq analysis. The ChIP‐seq data for H3K4me3 in spermatocytes (pachytene), round spermatids (Roundsp), testis and liver were analyzed as described in materials and methods, the H3K4me3 signal is represented in green color. The light gray lines and rectangles represent the structure of the murine *Catsper2* gene, and the first exon and part of the first intron of the divergent *Pdia3* gene. Red arrow marks the transcription start site.

The potential *Catsper2* promoter region was analyzed using The Eukaryotic Promoter Database. Two putative promoters of 601 and 784 bp were found, between −1289 to −689 and −500 to +284 relative to +1 transcription start site, respectively. The putative *Catsper2* promoter sequence containing the 1598 bp region (−1309 and +289) was obtained from the National Center for Biotechnology Information (NCBI) and further analyzed to search for regulatory elements. The promoter region near *Catsper2* contained neither a TATA consensus box nor a CCAAT consensus box near the TSS. In addition, a CpG island from −536 to +189 was detected with the methprimer software (Fig. 1A). Several transcription factor binding sites were predicted using promo and tf‐bind, including SOX, CTCF and CRE sites; only CRE and CTCF binding sites were found in the 784 bp promoter around the TSS (Fig. 1A).

CpG islands are regions of DNA with a high‐GC content and high frequency of CpG dinucleotides that have been strongly correlated to promoter regions and transcription initiation [32, 33]. The methylation status of the CpG island in the *Catsper2* promoter in testis and other tissues was evaluated through WGBS data analysis; as shown in Fig. 1B, the DNA of the region containing the murine *Catsper2* promoter is found non‐methylated in both spermatogenesis cells and liver and spleen tissues, which agrees with previous reports indicating that CpG islands associated with promoter regions do not usually show a tissue‐specific methylation pattern [34].

The histone mark H3K4me3 has been associated with a signature of a transcriptionally active promoter region [35, 36, 37]. To clarify whether the predicted *Catsper2* promoter region was functional *in vivo*, we performed H3K4me3 ChIP‐seq data analysis in mouse germ cells at different stages of spermatogenesis and whole testis and liver as controls (see Table 2 and Fig. 1C). As expected, we found the H3K4me3 modification enriched near the TSS of germ cells and whole testis, suggesting that the predicted promoter region has transcriptional activity. Nonetheless, we also observed the presence of this histone modification in liver tissue where *Catsper2* is not expressed. This fact can be attributed to the proximity of the *Pdia3* transcription start site, which is a ubiquitous gene [38, 39]. Thereby, the *Catsper2* promoter region lying from −1309 to +289 was selected for further transcriptional analysis.

### Transcriptional activity of the putative murine *Catsper2* promoter regions

**Fig. 2:**
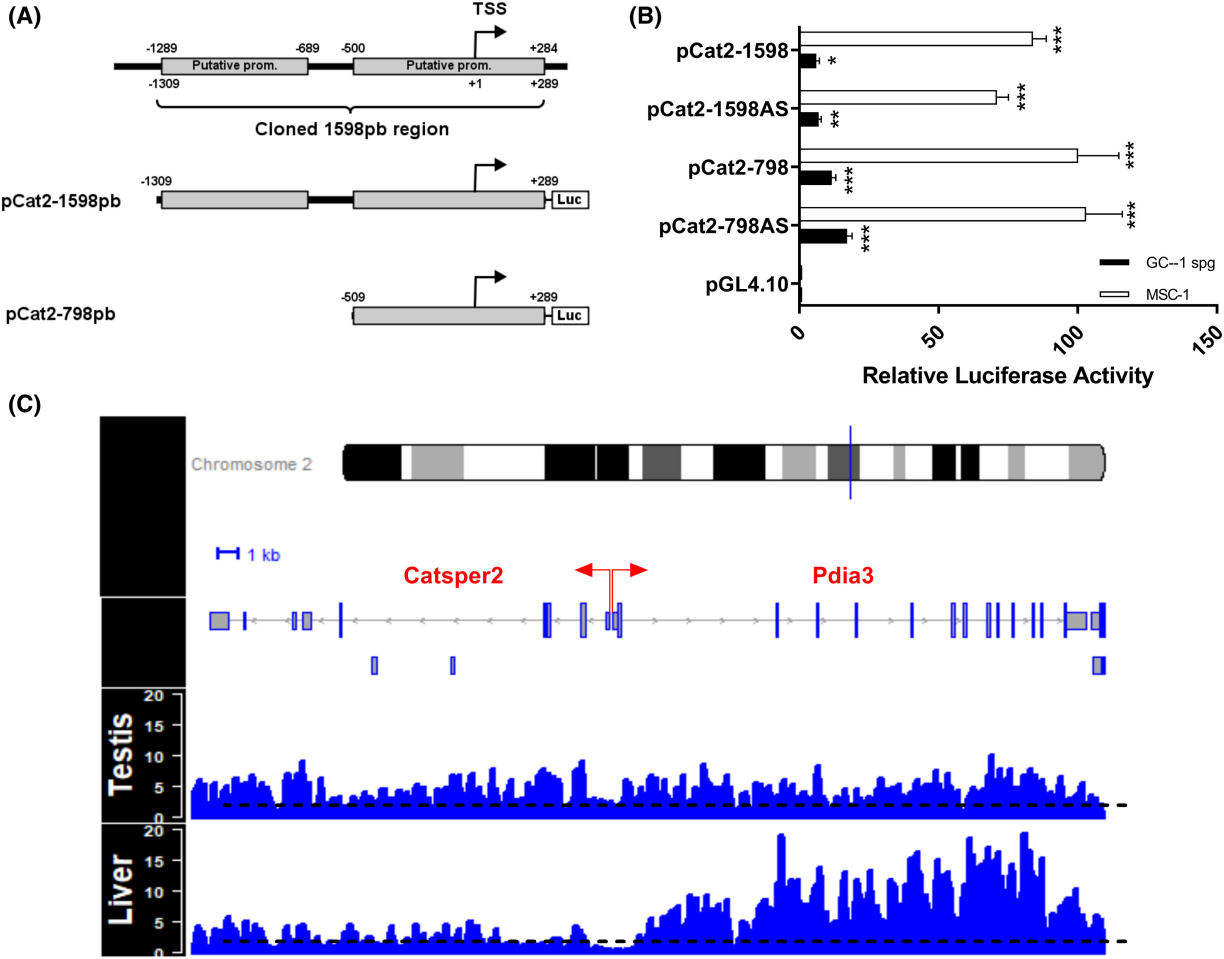
Transcriptional activity of the mouse *Catsper2* promoter. (A) a 1598 bp region containing the two predicted promoters and a 798 bp region containing only the putative promoter including the TSS (+1) were inserted upstream of the luciferase gene into vector pGL4.10[Luc2] (pCat2‐1598 and pCat2‐798, respectively). (B) both constructs were transiently transfected into the MSC‐1 and GC‐1 spg cell lines and the cells were harvested for luciferase assays after 48 h. these two regions were cloned in antisense (pCat2‐1598AS and pCat2‐798AS) and used as negative controls. Luciferase activities represent fold‐increments relative to the empty vector activity and are expressed in relative units, defined as the ratio of *Photinus* luciferase to *Renilla* luciferase activities. Data represent mean ± SEM (*n* = 9) of three independent experiments, each performed in triplicate. One‐way ANOVA was used to identify significant differences (*P < 0.05, **P < 0.01 and ***P < 0.001). (C) Histone H3K36 trimethylation patterns at the mouse *Catsper2*‐*Pdia3* locus based on the ChIP‐seq analysis. The ChIP‐seq data for H3K36me3 in 8‐week‐old mouse testis and liver for the region comprising the *Catsper2* and *Pdia3* genes were plotted. The light gray lines and rectangles represent the structure of the murine *Catsper2* and *Pdia3* genes. The black dashed lines show a normalized 2‐fold change from the input samples of each tissue. Red arrows mark the transcription start sites.

The fragments containing the putative promoters, the 1598 bp (from −1309 to +289) and 798 bp (−509 to +289) regions were cloned upstream of the luciferase reporter gene in pGL4.10 vector (pCat2‐1598 and pCat2‐798, respectively, Fig. 2A) to analyze the transcriptional activity of the *Catsper2* promoter. Both constructs were transiently transfected into GC‐1 spg and MSC‐1 cells and luciferase activities were then measured. Luciferase assays showed a 6.1‐ and 83.9‐fold increase in the promoter activity for pCat‐1598 compared to the promoterless vector pGL4.10 in GC‐1spg and MSC‐1, respectively. Meanwhile, the 800 bp 5′ deletion (pCat2‐798) revealed a transcriptional activity of 11.7‐ and 100.2‐fold increase in GC‐1spg and MSC‐1, respectively (Fig. 2B). These results indicate that the −509 to +289 region of *Catsper2* (pCat2‐798) contains a functional promoter. In addition, pCat2‐1598AS and pCat2‐798AS constructs containing putative *Catsper2* promoters in antisense also exhibited a transcriptional activity. The first construct showed an increase of 6.9 and 71.0 in GC‐1 spg cells and MSC‐1, and the second 17.2 and 103, respectively, suggesting that the divergent *Pdia3* gene promoter overlaps with *Catsper2* promoter or alternatively, *Catsper2* promoter may be bidirectional.

The histone mark H3K36me3 has been widely studied and related to transcriptional elongation since this mark can be detected on the body of transcriptionally active genes [40]. In addition, H3K36me3 modification may be one of the ways transcription is regulated in differentially expressed bidirectional promoters [41]. To further investigate the possible bidirectionality of the *Catsper2* promoter, previously reported ChIP‐seq data from testis and liver for the histone mark H3K36me3 were plotted (see Table 2 and Fig. 2C). Histone H3K36me3 mark was found throughout *Catsper2* gene in testis, similar to *Pdia3*, while a stronger H3K36me3 signal is observed in *Pdia3* gene in the liver.

### Delimiting *Catsper2* core promoter

**Fig. 3:**
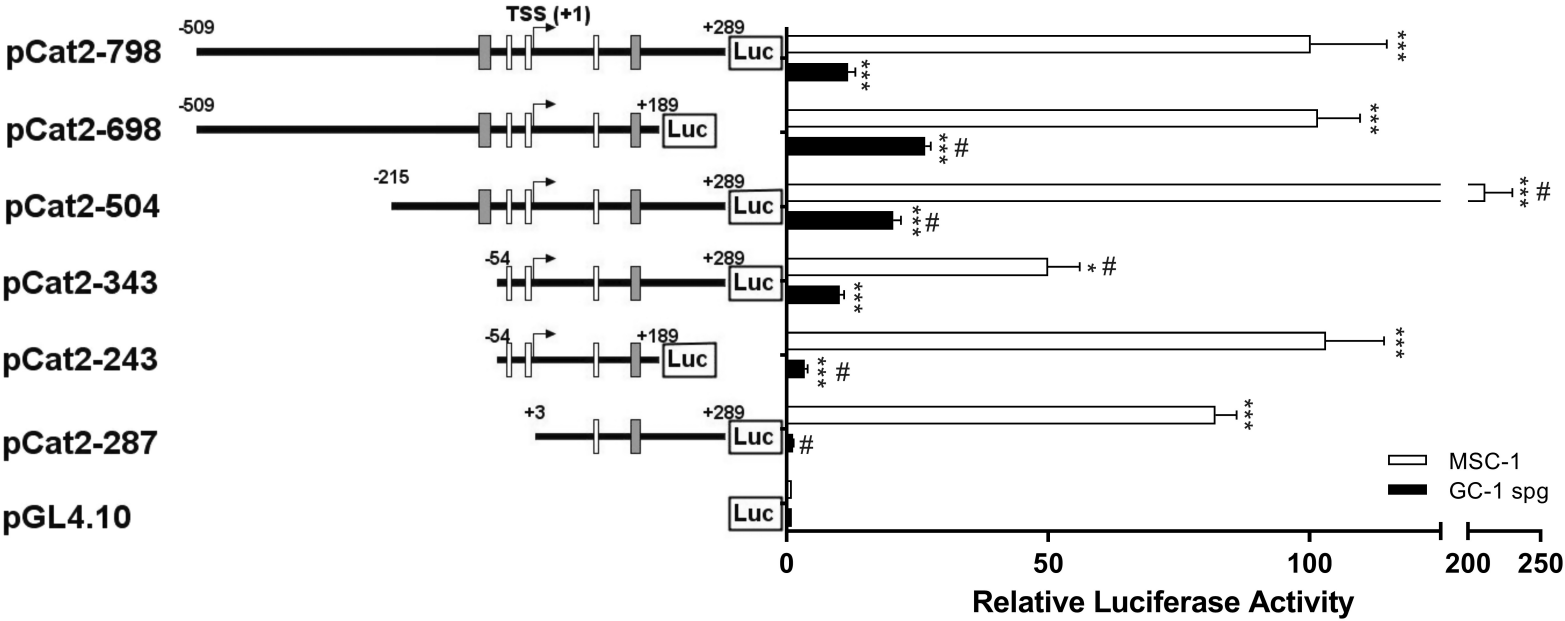
Deletion analysis of mouse *Catsper2* promoter. Plasmids with deletions at the 5′, 3′ or both ends of the *Catsper2* promoter were co‐transfected into GC‐1 spg and MSC‐1 cells using pRL‐CMV vector as a control of transfection efficiency. The positions are numbered according to TSS (+1), potential CRE and CTCF sites are indicated by gray and white boxes, respectively. Luciferase activities were calculated as in Fig. 2. Data are presented as the mean ± SEM (*n* = 9) of three independent experiments, each performed in triplicate. *Significant differences compared to the activity of pGL4.10. #Significant differences compared to pCat2‐798. One‐way ANOVA was used to determine statistical differences (***P < 0.001).

Deletion fragments were generated by PCR from the pCat2‐798 construct and subcloned into promoterless pGL4.10 reporter vector to determine the *Catsper2* basal promoter sequence with transcriptional activity. Deletion constructs were transfected into GC‐1 spg and MSC‐1 cells, and the transcriptional activity was measured by the luciferase activities of the reporter gene. The 100 bp deletion at the 3′ end (pCat2‐698) generated a 2.3‐fold increase in transcriptional activity in GC‐1 spg cells compared to the pCat2‐798 construct, and no change was observed in MSC‐1 cells (Fig. 3). In addition, a 5′ deletion of 294 bp (pCat2‐504) showed a 1.7‐ and 2.1‐fold increase in transcriptional activity both in GC‐1 spg and MSC‐1 cells, respectively, compared with pCat2‐798. Interestingly, as the 5′ end was further shortened to −54, the transcriptional activity for the pCat2‐343 construct decreased 1.1‐ and 2‐fold in GC‐1 spg and MSC‐1 cells, respectively. For the pCat2‐243 construct, a 3.3‐fold decrease was observed in GC‐1 spg cells compared with pCat2‐798. However, plasmid pCat2‐243 still showed statistically significant activity, a 3.5‐fold increase in GC‐1 spg cells compared with the empty vector, in contrast to the null transcriptional activity of the pCat2‐287 construct lacking TSS in GC‐1 spg cells. Deletion of TSS (pCat2‐287) did not abolish the transcriptional activity in MSC‐1 cells, which may be due to an alternative TSS. In addition, the difference in transcriptional activity of the *Catsper2* promoter in MSC‐1 and GC‐1 spg lines should be the result of the relative levels of the repressing and activating factors of the CREM and CREB families expressed in these cell lines considering that both families of transcription factors bind to CRE sites with the same affinity [42, 43]. These results suggest that the minimal *Catsper2* promoter sequence necessary for basal transcriptional activity both in GC‐1 spg and MSC‐1 cells lies between −54 and +189 relative to the TSS.

### CTCF and CREMτ enhance *Catsper2* promoter transcriptional activity

**Fig. 4:**
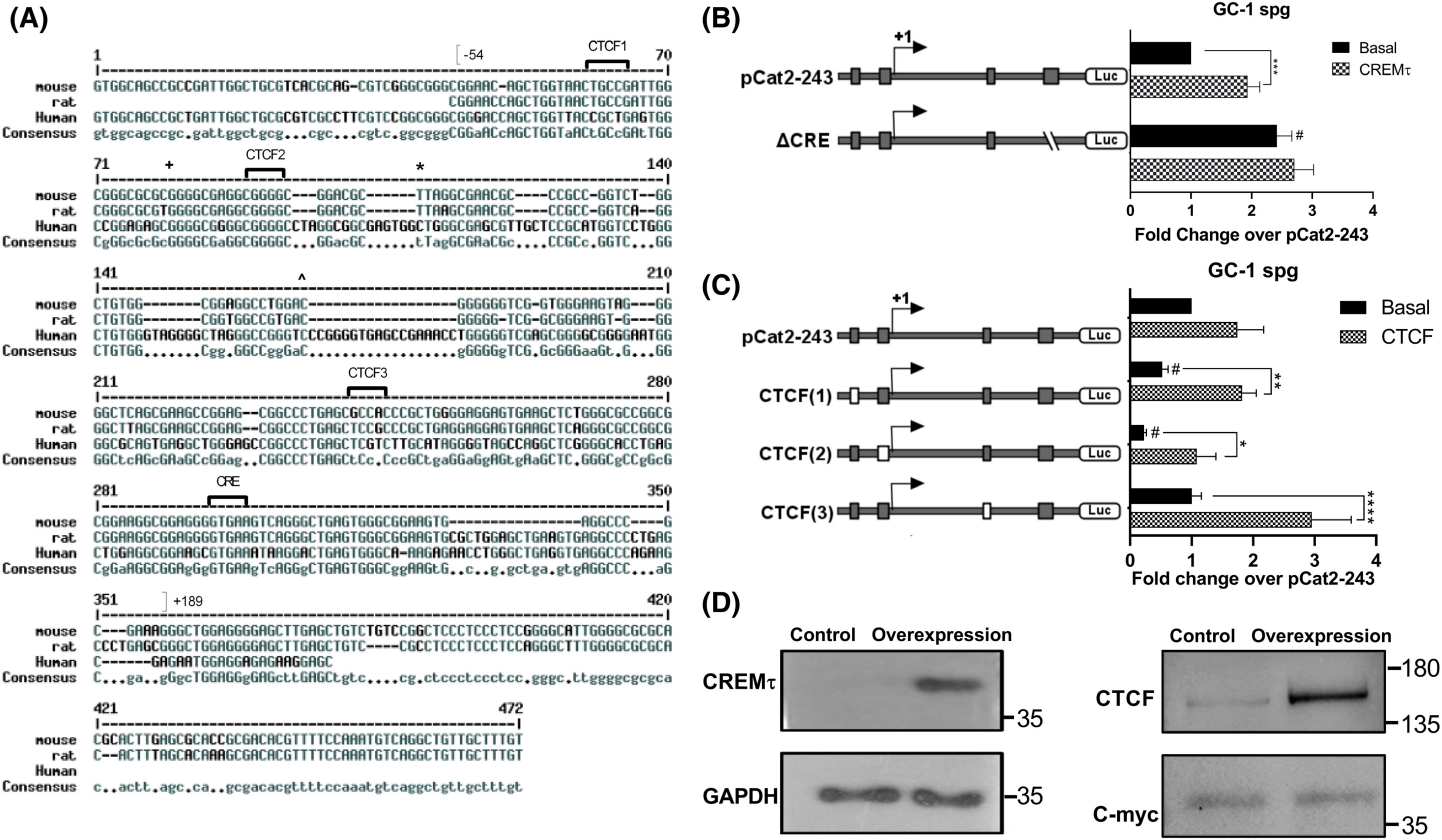
CRE and CTCF sites are conserved in mouse, rat, and human; these factors regulate *Catsper2* promoter activity in GC‐1 spg. (A) the sequences of the promoter regions of the mouse, rat and human *Catsper2* gene were aligned by ClustalX; putative CTCF and CRE binding sites are labeled and the NCBI‐predicted TSS of human (+), mouse (*) and rat (^) Catsper2 genes and the coordinates of the murine minimal promoter are indicated. (B) Plasmids pCat2‐243 and derived mutants ΔCRE or (C) CTCF (1), CTCF (2), CTCF (3) were transiently co‐transfected with pCREMτ (pcDNA3‐Cremτ) or pCTCF (pCMV6‐CTCF), respectively, into GC‐1 spg cells and the cells were harvested for luciferase assays after 48 h. the luciferase activities represent the fold change over pCat2‐243 construct. Data indicate mean ± SEM (*n* = 9) of three independent experiments, each performed in triplicate. Paired student *t*‐test was used to detect significant differences (*P < 0.05, **P < 0.01, ***P < 0.001 and ****P < 0.0001). ^#^Significant differences compared to pCat2‐243 basal activity. (D) Total protein from GC‐1 spg cells transfected with the constructs containing the Ctcf and Cremt genes (overexpression) and non‐transfected (control) was analyzed by western blot. Upper panels, endogenous and recombinant proteins were detected with specific antibodies (anti‐CREM and anti‐CTCF); lower panels, GAPDH and c‐MYC proteins were detected as loading control. Experiments of transfection were performed by triplicate.

The *in silico* analysis of the minimal *Catsper2* promoter region (−54/+189) indicates the presence of three CTCF sites (CTCF1, CTCF2, and CTCF3) and one CRE binding site (Fig. 1A). Furthermore, CTCF and CRE sites are conserved in mouse, rat, and human species, as shown in the multiple sequence alignment of this region (Fig. 4A), suggesting these TFs regulate *Catsper2* promoter. To ascertain the role of CTCF and the CREMτ in the transcriptional activity of *Catsper2* promoter, three CTCF sites were mutated (CTCF1‐3), and a CRE site (ΔCRE) was deleted in the core promoter of *Catsper2* (pCat2‐243) by site‐directed mutagenesis (Fig. 4B,C), and the promoter activity was evaluated in GC‐1 spg cells. To further assess the relevance of CTCF and CREMτ in *Catsper2* regulation, plasmids containing *Ctcf* or *Cremτ* ORFs were co‐transfected with the wild‐type or mutant promoter constructs. Overexpression of recombinant proteins in GC‐1 spg cells was verified by western blot with specific antibodies (Fig. 4D).

Overexpression of CREMτ or CTCF in GC‐1 spg cells provoked a 1.93‐ and 1.74‐ fold increase in the core promoter (pCat2‐243) transcriptional activity, respectively (Fig. 4B,C), suggesting that these transcription factors regulate murine *Catsper2* promoter. Likewise, deletion of the CRE site caused a 2.41‐fold increase over pCat2‐243 promoter activity, suggesting a repressive role for this site in GC‐1spg cells. The enhanced activity of the ΔCRE mutant was not changed by overexpression of CREMτ, which may be due to the deletion of the unique CRE target site (Fig. 4B). Regarding the CTCF binding sites, mutation of CTCF1 and CTCF2 reduced pCat2‐243 transcriptional activity (1.92‐ and 4.35‐fold, respectively), and no change was observed with CTCF3 mutant. CTCF overexpression increased the transcriptional activity of all three mutants and the CTCF3 mutant site showed the greatest activity (2.95‐fold change) relative to the wild‐type promoter construction (Fig. 4C). These results suggest that CTCF1, CTCF2, and CRE sites participate in the transcriptional regulation of *Catsper2* core promoter.

### CTCF and CREMτ bind to *Catsper2* core promoter *in vivo*

The aforementioned results suggest that CTCF and CREMτ regulate *Catsper2* promoter activity, possibly by binding to their specific sites in the core promoter. Therefore, ChIP assays were performed to assess the interaction of CTCF and CREMτ with *Catsper2* core promoter *in vivo*. Fragmented DNA from testes or liver chromatin was precipitated with anti‐CTCF, anti‐CREM or irrelevant anti‐MHC Class II (IgG) and then subjected to PCR.

**Fig. 5:**
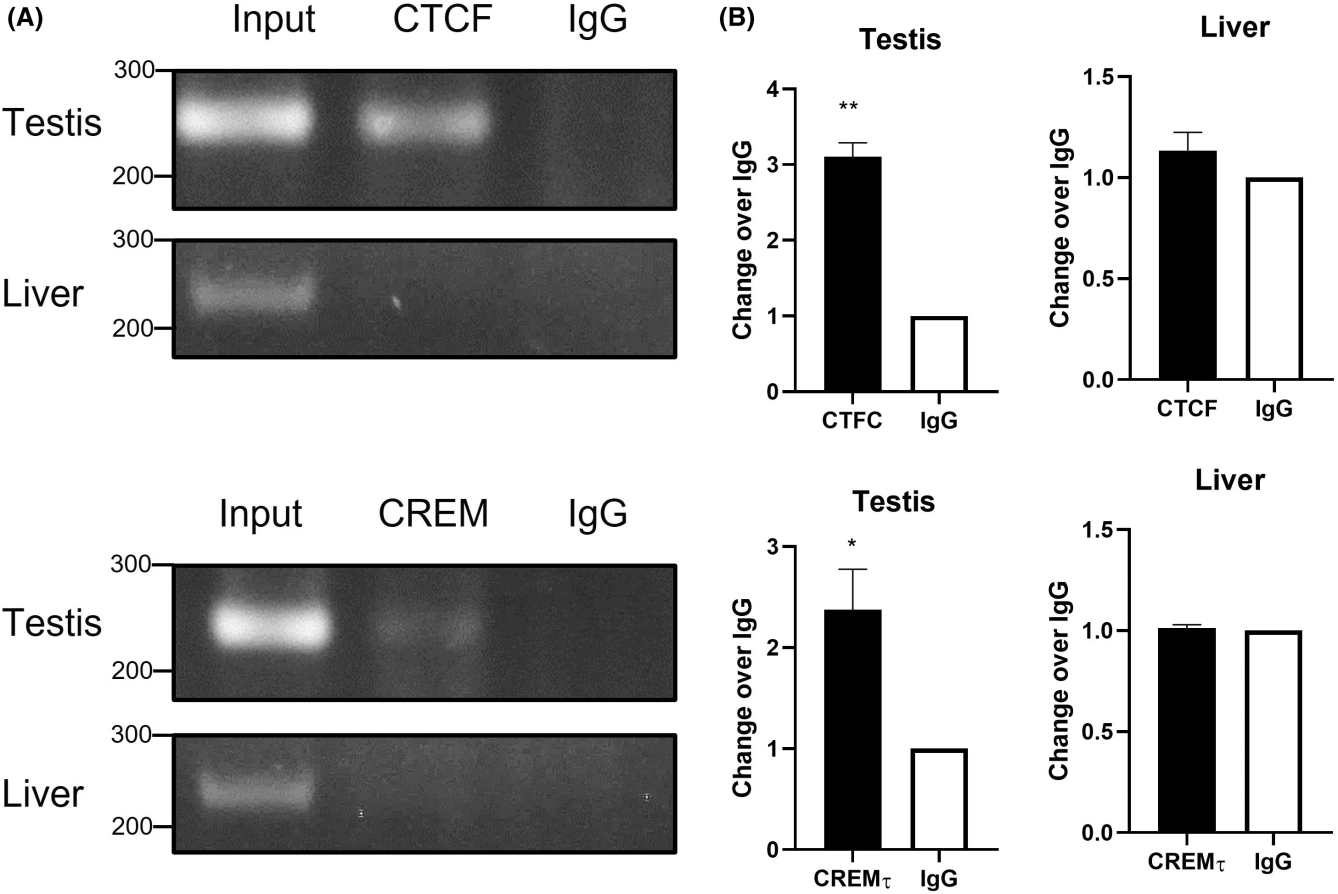
CREMτ and CTFC bind to murine *Catsper2* promoter *in vivo*. (A) ChIP‐PCR products were amplified with Cats2F/−54 and Cats2R/+189 primers for CREMτ and CTCF. Fragmented testicular chromatin was immunoprecipitated with anti‐CREM or anti‐CTCF antibody. MHC‐II antibody was used as a negative isotype specific (IgG) control, while liver chromatin as a non‐specific target binding control. Input DNA samples represent total chromatin. PCR products were separated in agarose gel (*n* = 3). (B) Densitometric analysis of CTCF and CREM ChIP‐PCR signals using imagej. Paired student *t*‐test was used to detect significant differences (*P < 0.05 and **P < 0.01).

ChIP results revealed the amplification of a specific region from *Catsper2* core promoter for both transcription factors in the testis samples. No signal was detected with the control MHC Class II antibody (Fig. 5). These results demonstrate that CTCF and CREMτ bind in a tissue‐specific manner to the murine *Catsper2* core promoter *in vivo*, corroborating their participation in *Catsper2* promoter regulation.

## Discussion

*Catsper2* gene, which codes for one of the main subunits of the CATSPER channel, is specific for spermatogenesis and essential for sperm hyperactivation and male fertility in both mice and humans [1, 11, 12, 13, 14, 15, 44]; The relevance of *Catsper2* gene in human male fertility has been evidenced by deletions or mutations in *Catsper2* gene associated with patients with deafness‐infertility syndrome (DIS) or non‐syndromic male infertility [11, 12, 13, 14, 15]; therefore, it is important to elucidate its transcriptional regulation mechanisms to explore whether there is a relationship with some cases of male infertility and to have a possible target in the development of male contraceptives. Here, we describe for the first time the murine *Catsper2* promoter region and some of the mechanisms that may be involved in its tissue‐specific expression. We found *in silico* the putative promoter in a region of approximately 1600 bp that includes the transcriptional start site (TSS +1); although, the transcriptional functional proximal promoter was delimited to a region of about 800 bp. An interesting finding was the transcriptional activity of the promoter region of *Catsper2* in antisense consistent with the head‐to‐head gene display of *Pdia3* in the mouse genome assembly GRCm39, which unlike *Catsper2* is a ubiquitous gene [38, 39]. Therefore, these results suggest that the promoter of the divergent *Pdia3* gene is found in the same region. Alternatively, *Catsper2* promoter may behave as a bidirectional promoter, as reported for some promoters whose genes are expressed in spermatogenesis [24, 45, 46].

H3K4me3 mark has been widely related to active promoter regions [35, 36, 37]. In this regard, a clear signal of the H3K4me3 mark over the predicted *Catsper2* promoter region in testis, late‐spermatocytes, and early spermatids was observed by ChIP‐seq data analysis. This evidence agrees with the reported *Catsper2* expression pattern in these cells [1, 44]; however, we also found the H3K4me3 signature in the liver. Therefore, the H3K4me3 mark keeps this chromatin region open so that transcription initiation occurs in the antisense direction for the ubiquitous *Pdia3* gene. In contrast, *Catsper2* transcription may be regulated by other epigenetic mechanisms as well as by specific transcription factors during spermatogenesis.

H3K36me3 is a histone mark, that has been related to transcription elongation and bidirectional promoters [40, 41]. Bidirectionality is consistent with the different expression patterns of *Catsper2*‐*Pdia3*, which appear to be regulated by the same promoter region since our luciferase assays showed that this region has transcriptional activity in both directions. According to ChIP‐seq data analysis, the presence of H3K36me3 on *Catsper2*, in contrast to *Pdia3*, is only found in testis, suggesting that H3K36me3 mark may be one of the factors contributing to the tissue‐specific expression of the *Catsper2* gene.

Unlike *Catsper1*, a CpG island was predicted by bioinformatic analysis in *Catsper2* promoter [47]. Most of the promoters contain CpG islands [48] and have been linked to TATA‐less promoters as is the case of the *Catsper2* promoter. Additionally, the histone methylation marks in *Catsper2* promoter region derived from ChIP‐seq data analysis are consistent with the genome‐wide studies that have associated promoter regions containing high CpG dinucleotide frequency with the H3K4me3 modification [33]. The same mark that has been proposed as one of the ways to prevent DNA methylation in these regions; this agrees with WGBS data analysis of the *Catsper2* promoter region.

Transcription factors also play a fundamental role in the regulation of gene expression [49]; and the predicted transcription factor binding sites of interest are concentrated in a region close to the TSS, which is related to the definition of a proximal promoter [50, 51]. We found that the minimal promoter of *Catsper2* comprises a 243 bp region (−54 to +189), including part of exon 1. Previously, the minimal promoter of murine *Catsper1* was found in a 394 bp region (−287 to +107), which also includes more than 100 bp of the first exon [47], suggesting that the 3′ region plays a relevant role in the regulation of *Catsper1‐4* gene promoters. Transcription factors regulating a promoter are usually conserved in different species [52]. Indeed, sequence alignment of the *Catsper2* minimal promoter region reveals that all four predicted TFs binding sites (one CRE and three CTCF) are conserved among mouse, rat and human species.

CRE sites are approximately 150 bp near the TSS in haploid germ cells, and 40% of these sites are known as half‐CRE since only half of the bases of the CRE consensus sites are found [43]; this is consistent with a half‐CRE site predicted in the *Catsper2* promoter (+152).

CREMτ activates various genes during spermatogenesis, including *Catsper1*; however, most are expressed in post‐meiotic stages [16, 53, 54, 55, 56, 57, 58, 59]. Repressor isoforms of the CREM protein (CREMα, CREMβ and CREMγ) are expressed in spermatogonia (GC‐1 spg) cells [42]; so, deletion of the only CRE site of the minimal promoter results in an increased transcriptional activity. On the other hand, overexpressing the activator isoform CREMτ does not further increase the minimal promoter's transcriptional activity, lacking the only target CRE site (ΔCRE). This agrees with the enhanced transcriptional activity of the wild‐type *Catsper2* minimal promoter under CREMτ overexpression, where CREMτ surpasses the endogenous repressor isoforms to bind CRE site. As spermatogenesis progresses and the levels of CREM repressor isoforms decrease, *Catsper2* transcription starts, and later, in the post‐meiosis stage, CREMτ can use the CRE site of the *Catsper2* promoter to increase its expression levels; however, further investigation needs to be done to confirm this hypothesis.

On the contrary, of all the CTCF sites in the genome, only 6% contain all four modules, 38% the core motif plus another module, and the rest only the core motif [60]. Mutation of CTCF1‐2 sites reduces *Catsper2* promoter activity, indicating that these sites are essential in regulating this promoter. According to the results of the mutant CTCF binding sites, CTCF2 is the most relevant site for Catsper2 minimal promoter since its mutation dramatically reduces the transcription and the specific factor fails to increase the activity to the levels of the wild‐type promoter sequence or mutant CTCF1 construct. Another interesting result to highlight is that CTCF3 site does not influence the basal activity of *Catsper2* minimal promoter, but for an unknown reason it prevents a greater transcriptional activation by CTCF protein through the CTCF1‐2 sites, leading to an enhanced activity of *Catsper2* promoter containing the mutant CTCF3.

In addition, the presence of CTCF‐binding sites has also been associated with promoters containing the H3K4me3 mark [61], demonstrating that these mechanisms are involved in the regulation of the *Catsper2* expression.

Finally, the regulatory mechanisms described here may be part of tissue‐specific regulation of *Catsper2* since the binding *of* both transcription factors to the basal *Catsper2* promoter only takes place in the testis according to the ChIP assay.

## Conclusions

Here, we show the first evidence for transcriptional regulation of *Catsper2* gene in the mouse model and spermatogonia cells. Our analysis suggests that the *Catsper2* promoter is under epigenetic regulation through H3K4me3 and H3K36me3 histone marks and a CpG island. Finally, our results show that this promoter region is regulated by the transcription factors CTCF and CREMτ; however, the exact molecular pathway and cellular stages have yet to be elucidated.

## Conflict of interest

The authors declare no conflict of interest.

## Author contributions

All the authors were involved in the overall study design. JH‐S, NO, MLB‐H, APC‐M, and SFL‐G conceived the experiments; APC‐M and SFL‐G conducted the experiments, analyzed the results, and drafted the manuscript; JH‐S, NO, APC‐M and SFL‐G reviewed the manuscript. All the authors have read and agreed to the published version of the manuscript.

## Data Availability

The data supporting this study's findings are available from the corresponding author (javierh@cinvestav.mx) upon reasonable request.
